# Pulmonary Involvement in a Mouse Model of Sjögren’s Syndrome Induced by STING Activation

**DOI:** 10.3390/ijms21124512

**Published:** 2020-06-25

**Authors:** Joanna Papinska, Harini Bagavant, Grzegorz B. Gmyrek, Umesh S. Deshmukh

**Affiliations:** Arthritis and Clinical Immunology Program, Oklahoma Medical Research Foundation, Oklahoma City, OK 73104, USA; Joanna-Papinska@omrf.org (J.P.); Harini-Bagavant@omrf.org (H.B.); Grzegorz-Gmyrek@ouhsc.edu (G.B.G.)

**Keywords:** Sjögren’s syndrome, STING, lung, interferon, salivary gland

## Abstract

Sjögren’s Syndrome (SS), a chronic autoimmune disorder affecting multiple organ systems, is characterized by an elevated type I interferon (IFN) response. Activation of Stimulator of Interferon Genes (STING) protein induces type I IFN and in mice, several features of SS, including anti-nuclear antibodies, sialadenitis, and salivary gland dysfunction. Since lung involvement occurs in one-fifth of SS patients, we investigated whether systemic activation of STING also leads to lung inflammation. Lungs from female C57BL/6 mice injected with the STING agonist 5, 6-Dimethylxanthenone-4-acetic acid (DMXAA), were evaluated for acute and chronic inflammatory responses. Within 4h of DMXAA injection, the expression of *Ifnb1*, *Il6*, *Tnf*, *Ifng*, and *Mx1* was significantly upregulated. At 1 and 2 months post-treatment, lungs showed lymphocytic infiltration in the peri-bronchial regions. The lungs from DMXAA treated mice showed an increased expression of multiple chemokines and an increase in lymphatic endothelial cells. Despite STING expression in bronchial epithelium and cells lining the alveolar wall, bone marrow chimeras between STING knockout and wild type mice showed that STING expression in hematopoietic cells was critical for lung inflammation. Our results suggest that activation of the STING pathway might be involved in SS patients with concomitant salivary gland and lung disease.

## 1. Introduction

Sjögren’s syndrome (SS) is a systemic autoimmune disorder marked by salivary and lacrimal gland dysfunction [1]. SS patients harbor circulating autoantibodies to intracellular proteins, including Ro60, Ro52, and La [2]. Salivary gland biopsies show lymphocytic foci in the peri-vascular and peri-ductal regions with larger inflammatory foci invading into the gland parenchyma [3]. The organization of the lymphocytic infiltrates into germinal centers increases the risk of lymphoma development [4]. Some patients show the destruction of glandular tissue, with fatty replacement, and extensive fibrosis [5]. Extraglandular manifestations of SS include the involvement of the joints, lungs, and the central and peripheral nervous systems [6,7].

SS patients show increased expression of type 1 interferon (IFN)-regulated genes (an IFN signature), which is associated with higher disease severity [8]. Our laboratory has established the pathogenic role of type 1 IFN in mouse models of SS [9,10,11]. We have previously reported that the production of type 1 IFN through activation of toll-like receptor 3 (TLR3) directly inhibits calcium mobilization in salivary gland cells [9]. Using mouse strains that spontaneously develop SS-like disease, we showed that repeated TLR3 stimulation can exacerbate salivary gland inflammation and dysfunction in NZB/W F1 mice [10] and that signaling through the IFNα/β receptor is required for SS development in B6. *Aec1Aec2* mice [11]. Viral infections are a potential mechanism for inducing IFN [12,13]. However, most SS patients fail to show evidence of recent or recurrent viral infections. Thus, the etiopathogenesis of SS is not entirely understood.

Recently, there has been an upsurge in the literature on innate immunity activation by cytosolic nucleic acid sensing pathways [14]. Nucleic acids, originating from exogenous (microbial) or endogenous (mitochondrial or nuclear) sources, bind nucleic acid receptors in the cytoplasm, and induce type 1 IFN and pro-inflammatory cytokine production. The Stimulator of Interferon Genes (STING) protein resides in the endoplasmic reticulum and is a central adaptor molecule in the cytosolic DNA sensing pathway [15]. STING binds to cyclic dinucleotide substrates and translocates to the ER Golgi intermediate compartment [16]. The subsequent recruitment of TBK1 and the phosphorylation of IRF3, which translocates to the nucleus, induces IFN production [17]. In addition, STING mediated activation of NF-κB results in the production of pro-inflammatory cytokines. Our previous work provides evidence that activation of the STING pathway initiates an SS-like disease in mice [18]. Female C57BL/6 mice injected with DMXAA, a cell-permeable agonist of murine STING [19], rapidly induced production of type 1 IFN and pro-inflammatory cytokines [18]. The mice subsequently developed anti-nuclear antibodies, salivary gland inflammation, and reduced salivary flow, thereby establishing a role for STING activation in SS pathogenesis.

In addition to exocrine dysfunction, pulmonary involvement occurs in up to 20% of SS patients, and it is a significant cause of mortality in SS [20,21,22]. However, a comprehensive evaluation with advanced imaging modalities like High-resolution CT scans and pulmonary lung function tests suggest a prevalence of subclinical disease in up to 58% of SS patients [23]. The reasons for why some SS patients show concomitant salivary gland and lung involvement are not known. In this study, to test the hypothesis that activation of innate immunity through the STING pathway induces lung pathology in SS, mice were injected with DMXAA, and the effects on lung were investigated.

## 2. Results

### 2.1. Systemic Activation of STING Leads to a Rapid Increase of Pro-inflammatory Gene Expression in the Lungs

**Figure 1:**
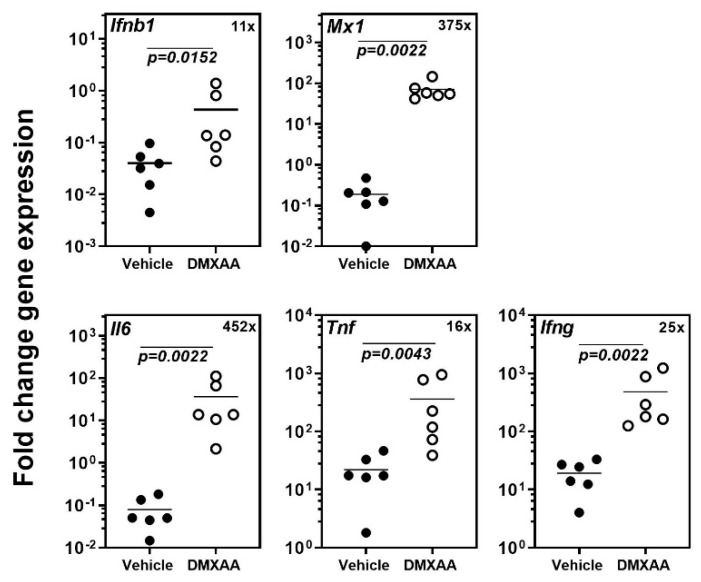
Systemic activation of the Stimulator of Interferon Genes (STING) pathway upregulates pro-inflammatory gene expression in the lungs. The expression of *Ifnb1*, *Il6*, *Tnf*, *Ifng*, and the type I IFN responsive gene *Mx1* in the lungs was significantly increased 4h after injection. The numbers in the top right corner represent fold increase (×) in gene expression over vehicle-treated mice. Statistical significance was determined by a two-tailed Mann–Whitney test, and *p* < 0.05 was considered significant (●: Vehicle-treated, ○: DMXAA-treated).

Our previous study demonstrated that systemic activation of STING with its agonist DMXAA caused a rapid and significant increase in circulating type I IFN and pro-inflammatory cytokines in mice [18]. Within 4 h, the expression of inflammatory cytokines and the type I IFN responsive gene *Mx1* was significantly elevated in the submandibular glands of mice. In the present study, to determine the acute effects of subcutaneous DMXAA injection on lungs, gene expression was evaluated by real-time PCR. A significant increase in the expression of *Ifnb1, Mx1, Il6, Tnf,* and *Ifng* was seen in the lungs (Figure 1). This increase in pro-inflammatory gene expression was comparable to the changes we have previously reported in the salivary glands of DMXAA injected mice [18].

**Figure 2:**
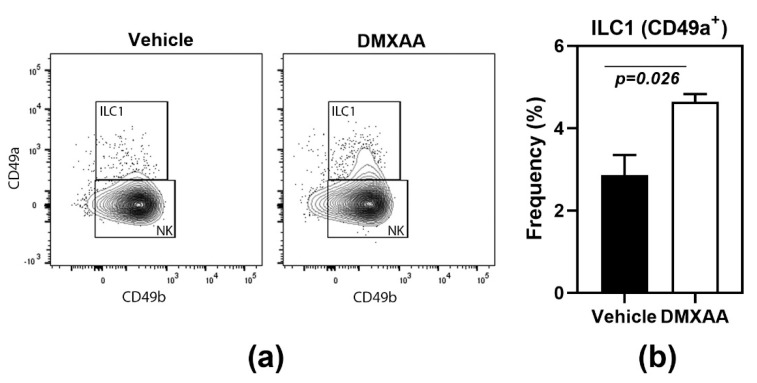
Increased frequency of type 1 innate lymphoid cells (ILC1) in lungs following STING activation. (**a**) The innate lymphoid cell populations were studied by flow cytometry. ILC1s were identified as CD49a+ cells in the CD45+ Lineage- NK1.1+ population; (**b**) On day 8, after injection, DMXAA-treated mice showed a significant increase in ILC1 cells in the lung. The ILC2 and ILC3 populations were not significantly different from vehicle-treated controls. Data shown are representative of two independent experiments with *n* = 3/group. The Mann–Whitney test was used to determine statistical significance.

Another feature shared with the salivary glands [18] was the significant increase of type 1 innate lymphoid cells (ILC1) in the lungs of DMXAA treated mice (Figure 2). Lungs of mice injected with DMXAA were evaluated by flow cytometry for frequencies of ILC1, ILC2, and ILC3. Of the different ILC populations, the ILC2 is the most dominant cell type in the lungs [24] and plays a crucial role in lung inflammatory responses [25]. However, DMXAA injected mice failed to show any changes in ILC2 populations in the lungs. Surprisingly, a small but significant increase in the frequency of ILC1 was seen on day 8 after injection (Figure 2).

Collectively, these data demonstrate that systemic STING activation by DMXAA has similar acute effects on the salivary glands and lungs.

### 2.2. Transient STING Activation Is Sufficient to Induce Lung Inflammation

**Figure 3:**
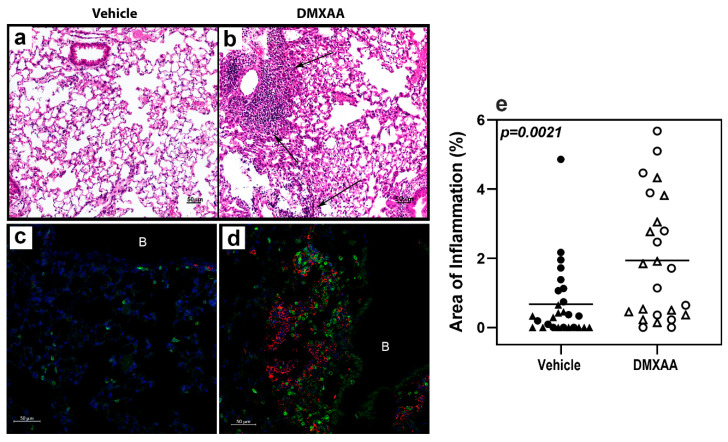
Lung inflammation in DMXAA treated mice. Representative micrographs of lungs from the vehicle (**a**,**c**) or DMXAA treated (**b**,**d**) mice one month after injection. Hematoxylin and eosin-stained sections (**a**,**b**) show inflammatory foci (arrows) in DMXAA treated mice. Immunofluorescence staining for immune cells in the lungs (**c**,**d**) show infiltrates composed of CD3+ T cells (green) and MHC II upregulation (red) in DMXAA-treated mice (**d**). B: Bronchial lumen, Scale bar = 50 μm. (**e**) Lung inflammation at one month (triangles) and two months (circles) after treatment. The two-tailed, Mann–Whitney test was used to determine statistical significance.

**Figure A1:**
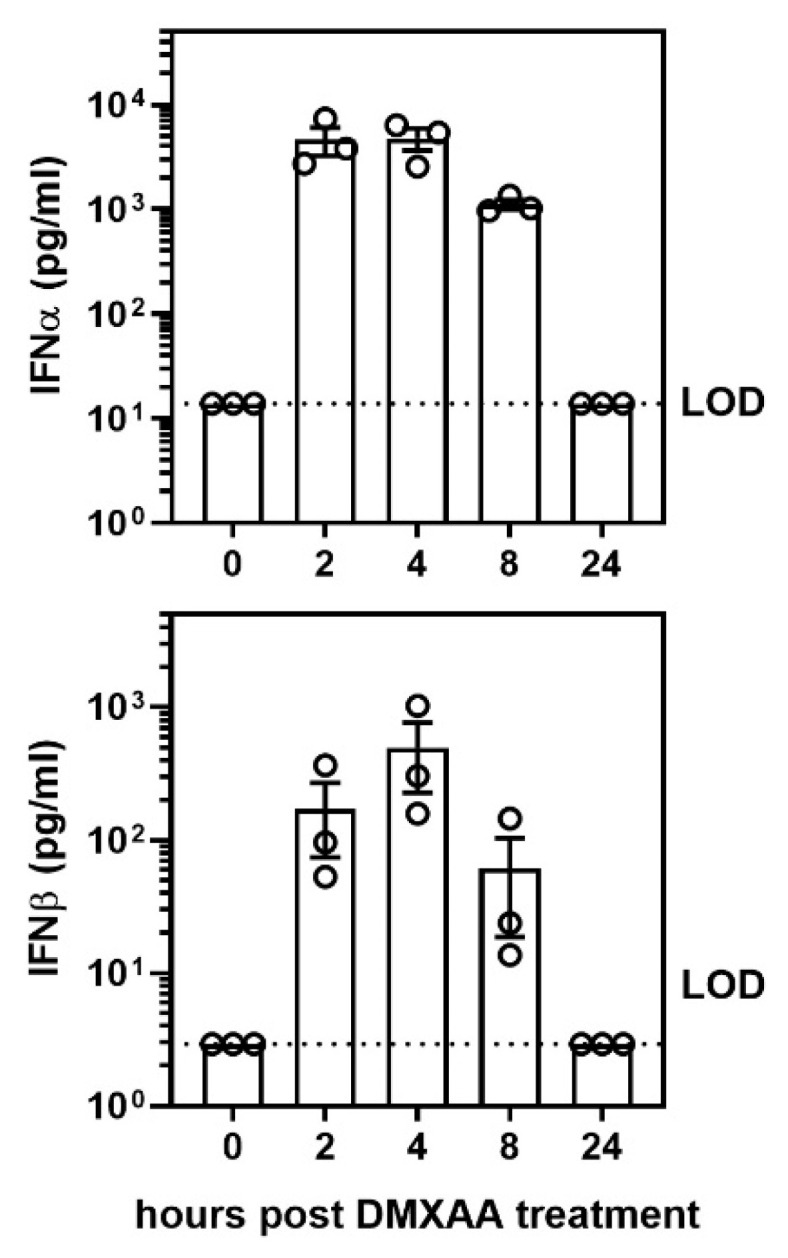
Activation of the STING pathway induced a rapid, albeit, transient elevation of IFNα and IFNβ cytokine production in serum after DMXAA injection. To determine the kinetics of systemic interferon production following STING activation, female mice were injected subcutaneously with DMXAA, and serum IFNα and IFNβ levels were measured 0, 2, 4, 8, and 24 h later. LOD: limit of detection. Each ○ represents data from an individual mouse.

After a subcutaneous DMXAA injection, there was a rapid increase in the serum IFNα and IFNβ levels. The circulating IFNα peaked at 2 h and IFNβ at 4 h after treatment (Figure A1). Interestingly, circulating IFN levels declined rapidly and were undetectable at 24 h. To determine whether this transient systemic response to STING activation had a long term effect on the lungs, mice were given two subcutaneous injections of DMXAA on days 0 and 21. Lungs were harvested one month after initial treatment and evaluated for inflammation. Lungs from DMXAA treated mice showed lymphocytic infiltration, predominantly in the peri-bronchial regions (Figure 3b). A few mice also showed small lymphocytic foci in the lung parenchyma. The inflammatory foci consisted primarily of CD3+ T cells intermingled with MHC II expressing antigen-presenting cells (Figure 3d). To determine whether this was a self-limiting inflammation, in some mice, the lungs were studied at 2 months after the first injection. The DMXAA treated mice had significantly higher lung inflammation compared to vehicle-treated mice (Figure 3e). The inflammation was present at 2 months, and there was no significant difference in severity scores in the DMXAA treated mice studied at the two different time points. Some of the vehicle-treated mice also showed small areas of lymphocytic infiltrates. Therefore, area of inflammation ≥ mean plus two standard deviations of vehicle-treated controls was considered as an indication of severe lung inflammation. Following DMXAA treatment, 9 out of 25 (36%) mice developed severe lung inflammation compared to only 1 out of 27 (3.7%) vehicle-treated control mice (*p* = 0.0042, Fisher’s exact test).

### 2.3. STING Activation Induces the Expansion of Lymphatic Endothelial Cells in the Lung

**Figure 4:**
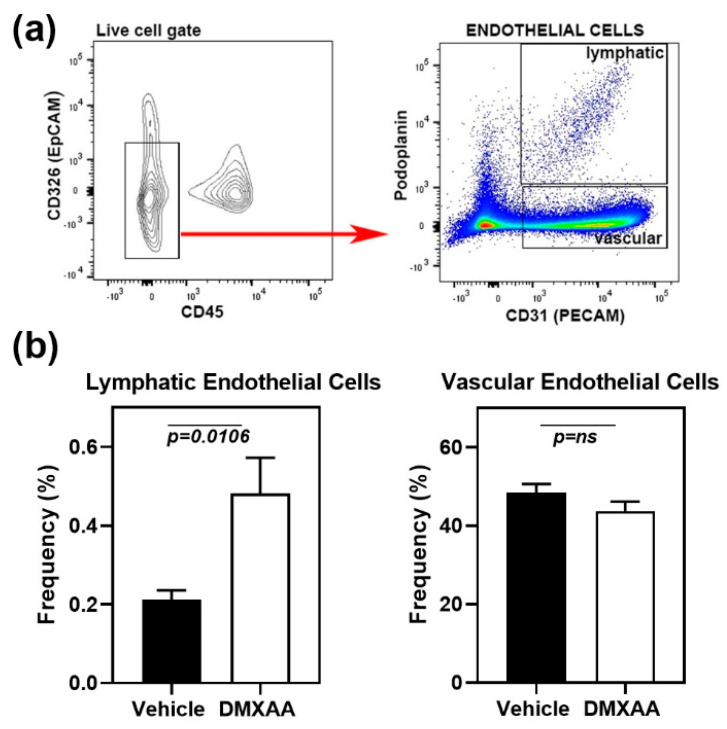
Expansion of lymphatic endothelial cells in the lungs of DMXAA treated mice. (**a**) **Left** Panel: Flow cytometry analyses showing CD45- CD326- endothelial cells (red arrow) in the live gate. **Right** panel: Gating strategy for lymphatic (CD31+ and Podoplanin+) and vascular (CD31+ and Podoplanin-) endothelial cells; (**b**) Lymphatic and vascular endothelial cells in lungs from DMXAA treated mice and vehicle-treated controls expressed as the frequency of the CD45- CD326- gate. Data are pooled from two independent experiments and represent mean + SEM of *n* = 9 mice/group. An unpaired two-tailed t-test was used to determine statistical significance, and *p* < 0.05 was considered significant.

Early lung involvement in SS patients is characterized by an increase in peri-bronchial infiltration [26] and the appearance of inflammatory cells in the bronchoalveolar lavage [27]. However, at later stages, the fibrotic changes in interstitial lung disease are the most common cause of morbidity [22]. Lymphatic vessel remodeling has been suggested to play an important role in pulmonary fibrosis [28]. In normal mouse lung, lymphatics are clustered along the larger airways, respiratory bronchioles, and intralobular arterioles. Intra-acinar arteries associated with alveolar ducts and near alveolar walls do not show the presence of lymphatic vessels [29]. To investigate whether the lung inflammation was associated with lymphatic changes, the frequencies of lymphatic endothelial cells (LEC) in the lung were studied by flow cytometry. Analysis of cell populations was carried out based on previously published literature [30]. The cell surface markers CD45 and CD326 (epithelial cell adhesion molecule) were used to exclude myeloid and epithelial cells, respectively (Figure 4a). LEC were identified as the CD31+ Podoplanin+ cell subset within the live CD45-CD326- gate. Vascular endothelial cells were defined as CD31+ Podoplanin- cell subset.

One month after DMXAA injection, mice showed a significant (*p* = 0.0106) increase in LEC frequencies compared to vehicle-treated controls (Figure 4b). LEC frequency and inflammation severity scores showed a significant (*p* = 0.0135), albeit a modest positive correlation (Pearson *r* = 0.57), suggesting that the expansion of LEC may be a consequence of inflammation. No change was seen in the vascular endothelial cells.

### 2.4. Lung Inflammation Is Associated with Changes in Inflammatory Gene Expression Profiles

**Figure 5:**
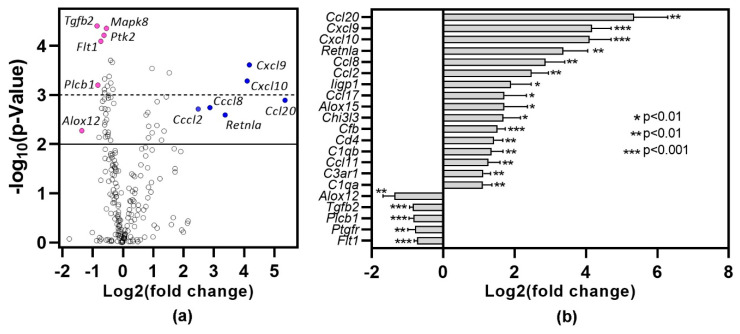
Differential gene expression profile in lung one month after DMXAA treatment. RNA from the lungs of DMXAA treated mice (*n* = 3 per group) and vehicle-treated mice (*n* = 5) were isolated and analyzed for expression using the nCounter Mouse Inflammation Panel (nanoString). Differential expression of genes was calculated by using the Advanced Analysis 2.0 module in the nSolver Software (nanoString). (**a**) Volcano plot of differentially expressed genes showing fold change (log2) versus *p*-value. The solid line denotes a *p* < 0.01, and the dotted line represents a *p* < 0.001. (●): Representative under-expressed genes, (●): Representative over-expressed genes; (**b**) Genes showing >2-fold increase and the 5 genes with the greatest drop in expression in DMXAA treated mice compared to vehicle-treated controls.

To gain insights into the pathways associated with inflammation, lungs harvested one month after DMXAA treatment were subjected to gene expression analysis using the nCounter mouse inflammation panel. Lungs from vehicle-treated mice were used as controls. Of the 254 genes in the inflammation panel that include 15 internal controls, 212 genes were expressed (Figure 5a). Of these, 89 genes showed statistically significant (*p* < 0.05) differential expression. Among the 16 genes showing at least a two-fold increase (Figure 5b), 50% belong to the chemokine family. *Ccl20* (40×, *p* = 0.0012), *Cxcl9* (18×, *p* = 0.0002) and *Cxcl10* (17×, *p* = 0.0005) were the top 3 differentially expressed chemokines. Resistin-like molecule alpha (*Retnla*), also known as Found in inflammatory zone 1 (*Fizz1*), is a well-established marker for M2 macrophages [31] and is upregulated during lung inflammation [32]. In DMXAA treated mice, *Retnla* expression showed a ten-fold increase (*p* = 0.0025). In our analysis, the only gene showing a two-fold decrease in expression was *Alox12*, whereas *Tgfb2* and *Plcb1* showed a 1.8-fold decrease.

### 2.5. STING Expression in Cells of Hematopoietic Origin Is Critical for Development of Inflammation in the Lungs

**Figure 6:**
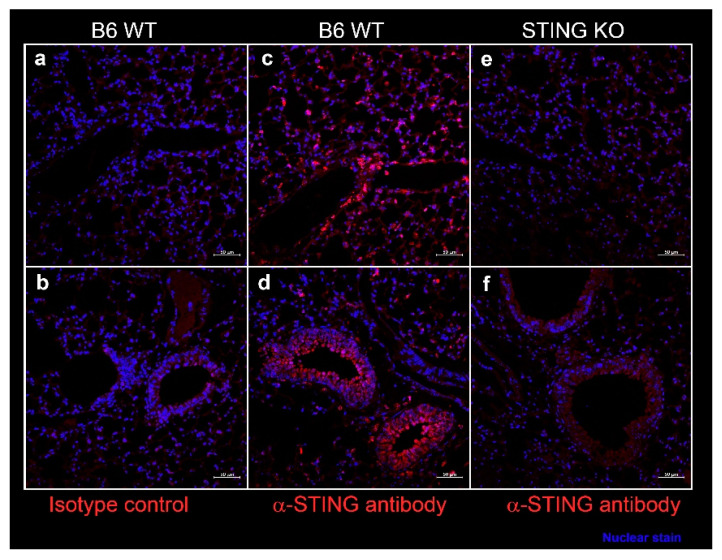
Expression of STING in the murine lung parenchyma (**a**,**c**,**e**), bronchi, and bronchioles (**b**,**d**,**f**). Formalin-fixed, paraffin-embedded lung sections from female C57BL/6J wild type (WT) mice and STING knockout mice at 10 weeks of age were stained with rabbit-anti-STING (**c**–**f**) or with isotype control rabbit IgG (**a**,**b**). Bound antibody was detected by AF647 conjugated-goat anti-rabbit IgG, and the images were captured on a Zeiss confocal microscope. STING expression (red) was seen in cells lining the alveoli (**c**) and in bronchial epithelium (**d**). Nuclei were stained with DAPI and appear blue. Scale bar = 50 μm.

In SS, epithelial cells play a considerable role in driving inflammation and dryness in mucosal linings. Since systemic DMXAA treatment induced an 11-fold increase in *Ifnb1* expression in the lungs (Figure 1), we entertained the possibility that STING activation in lung epithelial cells might contribute to this pro-inflammatory response. We evaluated STING expression by immunostaining of lung sections from wild type (WT) C57BL/6 mice (Figure 6). Intense staining for STING was seen along the alveolar walls in the lung parenchyma (Figure 6c) and the bronchial epithelial cell cytoplasm (Figure 6d). The specificity of STING localization was confirmed by the absence of staining with the anti-STING antibody in lung sections from STING KO mice (Figure 6e,f). No staining was seen when an isotype control was used as a primary antibody on WT lung sections (Figure 6a,b).

**Figure A2:**
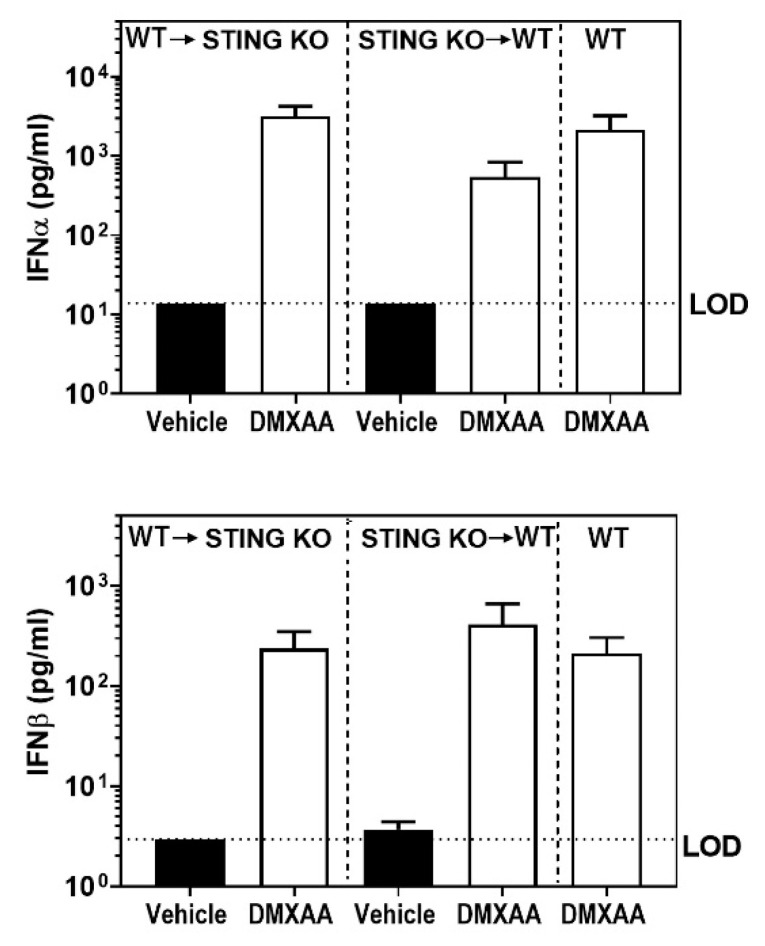
DMXAA induced IFNα and IFNβ production in bone marrow chimeras is comparable to wild type control mice. Bone marrow chimeric mice were injected with vehicle (solid bars) or DMXAA (open bars) and serum IFNα and IFNβ cytokine levels were determined 4h after DMXAA injection. WT mice injected with DMXAA were used as controls. Results are represented at mean ± SEM pooled from two independent experiments *n* = 8–10 mice/group. LOD: limit of detection.

To determine the role of STING activation in hematopoietic versus non-hematopoietic cells, bone marrow chimeras were generated between STING knockout and WT mice. Recipient mice were subjected to a lethal dose of irradiation followed by transfer of donor bone marrow cells [33]. Eight weeks following engraftment, mice were injected with DMXAA, and serum levels of IFN were measured (Figure A2). Following DMXAA treatment, both sets of bone marrow chimeras produced IFNα and IFNβ. Surprisingly, the IFNα and IFNβ levels were comparable to the DMXAA treated un-irradiated WT control mice (Figure A2). These data suggest that both hematopoietic and non-hematopoietic cells contribute to IFN production.

**Figure 7:**
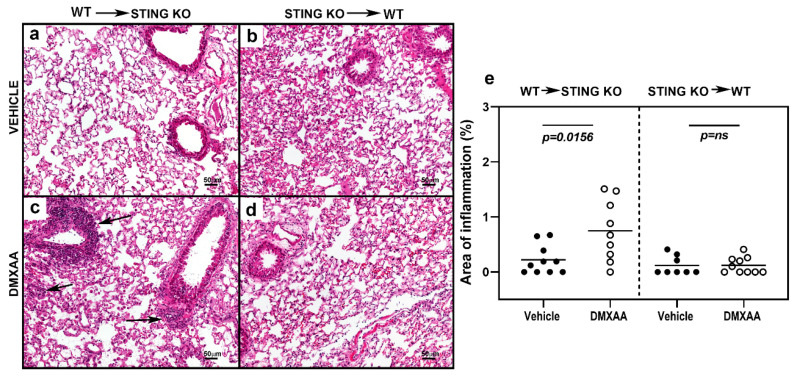
STING expression in the hematopoietic cells is critical for the development of lung inflammation in DMXAA treated mice. Representative images of hematoxylin and eosin-stained lung sections from bone marrow chimeras one month after injection with vehicle (**a**,**b**) or DMXAA (**c**,**d**). Lung inflammation (arrows) was seen following DMXAA treatment in STING KO mice reconstituted with WT bone marrow. Scale bar = 50 μm. (**e**) Lung inflammation in vehicle and DMXAA treated chimeric mice one month after injection. Results are pooled from two independent experiments. Two-tailed, Mann–Whitney test was used to determine the statistical significance, and *p* < 0.05 was considered significant (●: Vehicle-treated, ○: DMXAA-treated).

To investigate chronic effects, mice were injected with DMXAA on days 0 and 21, and studied for lung inflammation one month after the first injection. Severe lung inflammation was only evident in DMXAA injected STING knockout mice transplanted with WT bone marrow (Figure 7c). The severity scores for lung inflammation in this group of mice were significantly higher than vehicle-treated mice (Figure 7e). Collectively, these results demonstrate that while both hematopoietic and non-hematopoietic cells contribute to STING induced IFN production, cellular inflammation in the organ requires STING expression on cells of hematopoietic origin.

## 3. Discussion

In this study, we have investigated pulmonary involvement in a mouse model of SS-like disease induced by the activation of the STING pathway. Our results show that subcutaneous injection of female C57BL/6 mice with DMXAA, a murine STING agonist, induces a rapid, albeit transient, type 1 IFN production by cells of hematopoietic as well as non-hematopoietic origin. Activation of the STING pathway induces an early upregulation of pro-inflammatory cytokines, and the recruitment of ILC1 into the lungs. The consequences of acute STING activation persist and lead to the formation of inflammatory foci within the lungs.

The aberrant activation of the STING pathway is seen in patients with gain of function mutations in the *TMEM173* gene that encodes for STING [34]. It manifests as STING associated vasculopathy with onset in infancy (SAVI), characterized by inflammation in the skin, blood vessels, and lungs. Lung inflammation in SAVI patients leads to interstitial lung disease and pulmonary fibrosis. Whether SAVI patients also develop salivary gland inflammation is not known. However, unlike this autoinflammatory condition, wherein there is a constant and chronic activation of STING, our study demonstrates that even transient activation of the STING pathway is sufficient to induce lung inflammation that persists for 2 months and possibly even beyond.

The analysis of inflammatory gene expression in the lungs at the 1-month time point demonstrated that three chemokines, *Cxcl9*, *Cxcl10*, and *Ccl20*, showed the highest differential expression between DMXAA and vehicle-treated mice. CXCL9 and CXCL10 bind the chemokine receptor CXCR3, which is mainly expressed on memory CD4+ and CD8+ and activated CD8+ T cells [35]. CCL20 binds CCR6, which is mainly expressed on T cells, B cells, and dendritic cells [36]. IFNγ upregulates the expression of CXCL9 (also known as MIG) and CXCL10 (also known as IP-10). Thus, one can envision a continual feed-forward loop of inflammation, wherein IFNγ production by the infiltrating T cells and innate immune cells leads to chemokine production by epithelial cells, which sustains infiltration and formation of inflammatory foci in the lungs. Interestingly, the expression of CXCL9 and CXCL10 is elevated in the salivary glands of SS patients [37]. It would be of interest to evaluate the expression of these chemokines in the bronchoalveolar lavage of patients as putative biomarkers for lung involvement in SS.

In our study, we noted an expansion of lymphatic endothelial cells, which is suggestive of lymphatic remodeling, and a potential precursor to fibrotic change. In SS patients, the analysis of minor labial gland biopsies shows the formation of tertiary lymphoid structures [38]. This process occurs with an organized remodeling of lymphatic vessels and increases in lymphatic endothelial cells [30]. Whether tertiary lymphoid structures and fibrotic changes occur in our model system at later time points and affect lung function needs to be determined.

Although exocrine gland dysfunction is a dominant feature of SS, a considerable number of patients (63–65%) have histories of or present with clinical features of other systemic involvement [39]. The factors that regulate disease in exocrine glands and different organs are not known. As we have previously reported, DMXAA treatment induces inflammation in the salivary and lacrimal glands [18]. However, except for the lungs, the evaluation of other organs, including liver, heart, kidney, and pancreas from DMXAA treated mice did not show pathologic changes. These data suggest that common pathways might be responsible for the co-existence of the salivary gland and lung inflammation in a significant number of SS patients. Due to the considerable involvement of salivary gland epithelial cells in disease pathogenesis, SS has also been defined as autoimmune epithelitis [40]. Whether lung epithelial cells in SS patients mimic features of salivary gland epithelial cells in influencing the disease processes needs to be investigated. Interestingly, a unique feature of epithelial cells from these two organs is the surface expression of TLR3 [41,42], which is otherwise an endosomal receptor for viral dsRNA [43]. We have previously shown that injection of mice with TLR3 agonist poly (I:C) induces type I IFN mediated salivary gland dysfunction and accelerates SS in mice [10]. In the lungs, poly (I:C) treatment triggers a robust inflammatory response, which may exacerbate lung diseases like asthma or chronic obstructive pulmonary disease [42]. Thus, similar responses to innate stimuli by salivary gland ductal epithelial cells and lung epithelial cells might predispose these organs as targets for immune-mediated injury in SS.

The reported presence of anti-Ro/SSA and anti-La/SSB in SS patients with interstitial lung disease is highly variable [44]. In a recent study, Gao et al. reported that almost 48% of primary SS patients with interstitial lung disease are negative for both anti-Ro/SSA and La/SSB [45]. In our model system, we did not detect antibodies to Ro/SSA and La/SSB [18]. Instead, several mice developed high-titer anti-nuclear antibodies (ANA). In this context, it is of interest to note that primary SS patients with ANA, but lacking anti-Ro/SSA and anti-La/SSB showed a higher prevalence of pulmonary involvement [46].

In this study, we have used DMXAA, a synthetic, cell-permeant agonist of murine STING as a tool to activate the STING pathway. The canonical ligand of STING, cyclic GMP-AMP (cGAMP), is generated when cyclic GMP-AMP synthase (cGAS) senses dsDNA in the cytosol. Upon dimerization of STING by cGAMP, a cascade of signaling events leads to IFN and pro-inflammatory cytokine production. In SS patients, several different mechanisms may lead to increased cytoplasmic DNA and activation of nucleic acid sensors. SS patients show reduced DNase 1 activity and high levels of cytosolic DNA in peripheral blood mononuclear cells [47]. Gamma-interferon inducible protein 16 (IFI16), a cytosolic DNA sensor, is elevated in SS patients [48], and it co-operates with cGAS to amplify the STING response [49]. Furthermore, oxidative stress-mediated mitochondrial damage is a potent stimulus for the release of mitochondrial DNA into the cytosol [50]. Considering our findings demonstrating that STING activation plays an essential role in the pathogenesis of SS, dampening the STING pathway might prove to be a novel therapeutic strategy for treating SS.

## 4. Materials and Methods

### 4.1. Mice

All mouse experiments were approved by the Institutional Animal Care and Use Committee (Protocol#18-36, approval date: September 2018), and were in accordance with the National Institutes of Health guidelines. C57BL/6J wild type (WT) mice were purchased from The Jackson Laboratory (Bar Harbor, ME, USA) and used for experiments. A breeder pair of STING knockout Goldenticket *Tmem173^gt^* mice [51] was purchased from The Jackson Laboratory (stock #017537). The STING knockout mice for experiments were generated in the vivarium at the Oklahoma Medical Research Foundation. SS predominantly affects women over men with a ratio of almost 9:1 [1,2]. Hence, in this study, only female mice at 10–12wks of age were used. DMXAA (TOCRIS, Minneapolis, MN, USA) dissolved in sterile endotoxin-free 5% sodium bicarbonate was injected subcutaneously at a dose of 20 mg/kg [18]. In the long-term experiments, mice were injected on days 0 and 21. Control mice were injected with 5% sodium bicarbonate alone at the corresponding time points. The mice were housed in specific pathogen-free conditions with constant access to food and water, and fed the 5053 PicoLab Rodent Diet 20. The mice were euthanized at different times, and lungs were processed for histopathology, immunostaining, RNA isolation, and flow cytometry.

### 4.2. Gene Expression Analysis

Analysis of gene expression in the lung was performed by real-time PCR as previously described for salivary glands [18]. For multiplex gene analysis, RNA was isolated and gene expression was studied using the nCounter Mouse Inflammation Panel (nanoString, Seattle, WA, USA). Differential expression of genes was calculated by using the Advanced Analysis 2.0 module in the nSolver Software (nanoString, Seattle, WA, USA).

### 4.3. Histopathology

Lungs were inflated, fixed in 10% buffered formalin, and processed for paraffin embedding. The right middle lobe was used for histopathologic analysis. Five-micron sections were stained with hematoxylin and eosin using standard methods. Images were scanned on an Aperio CS2 digital pathology scanner (Leica Biosystems, Buffalo Grove, IL, USA). An observer blinded to the experimental details quantified the images for areas occupied by lymphocytic infiltrates and area of the entire section by using the Aperio ImageScope software. The results of lung pathology are expressed as (area of inflammation/total area) × 100 for each mouse. On an average 6.4 ± 0.548 (mean ± SEM) mm^2^ of total lung tissue was studied for each mouse.

**Figure A3:**
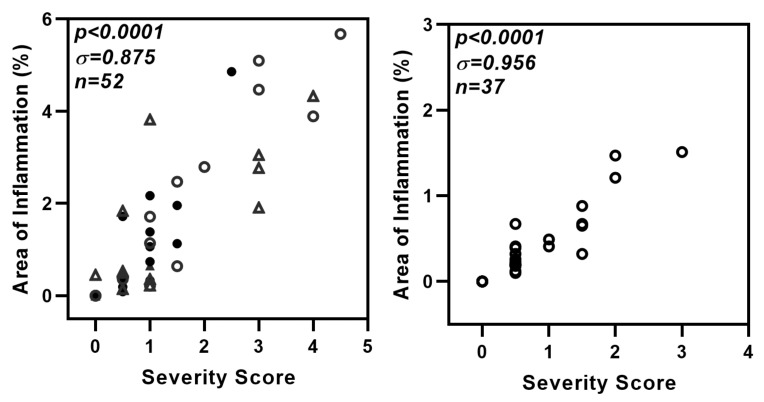
Strong correlation between two methods used for the evaluation of lung inflammation. The hematoxylin and eosin-stained lung sections (used in Figure 3e and Figure 7e) were also evaluated manually for severity of lung inflammation by an observer blinded to the experimental details. Lung pathology was evaluated for extent of airway and parenchymal involvement and severity of lymphocytic infiltration. The inflammation was scored on a scale of 0–5 with 0 = no inflammation and 1–5 representing increasing severity. A strong correlation is seen between data presented as % area of inflammation versus severity score. Left panel: comparison for data in Figure 3e. (△ and ○: DMXAA-treated at one and two months respectively; ▲ and ●: Vehicle-treated at one and two months respectively.) Right panel: comparison for data in Figure 7e (Each circle (○) represents data point from one mouse).

As an additional method, lung sections were also scored manually (Figure A3). Lung pathology was evaluated for extent of airway and parenchymal involvement and severity of lymphocytic infiltration [52]. The inflammation was scored on a scale of 0–5 with 0 = no inflammation and 1–5 representing increasing severity. An observer blinded to the experimental details read all slides.

### 4.4. Immunofluorescence Staining

Immunostaining for STING expression was carried out as previously described, with some modifications [18]. Briefly, formalin-fixed, paraffin-embedded, lung sections were deparaffinized and rehydrated in a decreasing alcohol gradient. Heat-induced epitope retrieval was carried out under acidic conditions. Slides were washed with PBS containing 0.1% Tween 20 and blocked with 10% normal horse serum in PBS. The sections were then incubated with a rabbit anti-STING antibody (5 μg/mL; Cell Signaling Technology, Danvers, MA, USA), overnight at 4 °C. Control sections were incubated with an equivalent concentration of purified rabbit IgG. After washing, the slides were incubated with donkey α-rabbit IgG-Alexa 647 conjugate (1:200, Jackson Immunoresearch Laboratories, West Grove, PA, USA) for 4 h at room temperature. All incubation steps were carried out in PBS with 1% bovine serum albumin as diluent and all washes were performed in PBS containing 0.1% Tween 20. Nuclei were stained with DAPI and Prolong Gold Antifade reagent (Thermo Fisher, Waltham, MA, USA) used as coverslip mounting media.

For evaluating the immune cells infiltrates, the right inferior lobe of the lung was inflated and fixed in periodate-lysine-paraformaldehyde fixative, transferred to 30% sucrose in PBS and embedded in OCT compound. Five-micron sections were stained with fluorochrome-conjugated antibodies to CD3 (145-2C11, Biolegend, San Diego, CA, USA) and MHC II (114.15.2, Biolegend) using standard methods [53]. Images were captured on a Zeiss LSM-710 confocal microscope.

### 4.5. Cytokine Measurements

IFNα and IFNβ levels in serum were measured using a magnetic bead-based ProcartaPlex Immunoassay (Thermo Fisher, Waltham, MA, USA) using manufacturers protocols.

### 4.6. Flow Cytometry

Lungs were digested with collagenase D to obtain single-cell suspensions, and stained with fluorochrome-conjugated antibodies using standard protocols [10]. Antibody panels used for innate immune cells [24] and lymphatic endothelial cells [30] are listed in Table A1. For all experiments, the right superior lobe was used to study lymphatic endothelial cells and the left lung was used to study innate immune cells. Zombie aqua fixable viability kit (Biolegend) was used for live/dead discrimination. Sample acquisition was performed using LSR II cytometer with Diva Software (BD Biosciences, San Jose, CA, USA) and analysis were carried out using FlowJo (Becton, Dickinson & Company, Franklin Lakes, NJ, USA).

### 4.7. Generation of Bone Marrow Chimeras

C57BL/6J wild type (WT) and STING KO mice were lethally irradiated (2 doses of 600 rads, 4 h apart) and injected with bone marrow cells from STING KO and WT recipients respectively, 4 × 10^6^ cells/mouse. The mice were allowed to recover and reconstitute the injected cells for 8 weeks before injection with DMXAA or vehicle.

### 4.8. Statistical Methods

Graph Pad Prism 8.0 (GraphPad Software, San Diego, CA, USA) was utilized to perform the statistical tests. Normality test was performed on each data sets. Parametric t test was used for datasets following normal distributions, and non-parametric Mann Whitney test was used for non-Gaussian distributions. All tests were two-tailed at 95% confidence interval and a *p*-value < 0.05 was considered significant.

## Abbreviations

IFN	Interferon
SS	Sjögren’s syndrome
STING	Stimulator of interferon genes
DMXAA	5,6-dimethylxanthenone-4-acetic acid
TLR3	Toll like receptor 3
ILC	Innate lymphoid cells
SAVI	STING-associated vasculopathy with onset in infancy
cGAS	Cyclic GMP AMP synthase
ANA	Anti-nuclear antibody

## Appendix A

**Table A1:** Antibody panels used for Flow cytometry.

Innate Lymphoid Cells			
Name of Antibody	Clone	Fluorophore	Manufacturer
Anti-mouse CD3ε	145-2C11	PE	eBioscience
Anti-mouse CD5	53-7.3	PE	eBioscience
Anti-mouse CD19	eBioID3	PE	eBioscience
Anti-mouse TCRγδ	eBioGL3	PE	eBioscience
Anti-mouse TCRβ	H57-597	PE	BD Biosciences
Anti-mouse FcεRI	MAR-1	PE	eBioscience
Anti-mouse CD8α	53-6.7	PE	eBioscience
Anti-mouse CD4	RM4-5	PE-Cy5	BD Biosciences
Anti-mouse Thy1.2 (CD90.2)	30-H12	PE-Cy7	eBioscience
Anti-mouse NK1.1	PK136	AlexaFluor780	eBioscience
Anti-mouse CD49a	Ha31/8	PerCp-Cy5.5	BD Biosciences
Anti-mouse CD49b	DX5	BV421	Biolegend
Anti-mouse CD45	30-F11	BV605	eBioscience
Anti-mouse GATA3	TWAJ	APC	eBioscience
Anti-mouse RORγT	Q31-378	BV786	BD Horizon
**Lymphatic Endothelial Cells**			
**Name of Antibody**	**Clone**	**Fluorophore**	**Manufacturer**
Anti-mouse CD45	30.F11	Pacific Blue	eBioscience
Anti-mouse CD326	G8.8	PE	BioLegend
Anti-mouse CD31	390	PE-Cy7	eBioscience
Anti-mouse Podoplanin	8.1.1	APC	Biolegend
